# Our New Department

**Published:** 1871-02

**Authors:** 


### PART III.

### Biographical, Ethical, Hygenic, and Miscellaneous.

### OUR NEW DEPARTMENT.

We call attention to this, our new department of the Companion. We will, from time to time, give biographical sketches of distinguished medical gentlemen. We hope these sketches will prove highly beneficial as indices to point the good and true of our profession to success and honor. We also propose to discuss medical ethics—the laws which should govern and control our action at all times, in our professional intercourse with one another—to show that he who will faithfully and conscientiously follow its teaching will, in the end, gain the esteem and confidence of the public. Principle, based upon truth, is the motive power that is sure to impel the good man upon the high road to success and honor. To depart from it, for present gain or selfish purposes, betrays a mind unstable and unwise; while such departure will, sooner or later, bring bitter fruits of repentance, disappointment and disgrace. As a public benefactor, the physician, to be able to meet the demands of his science to the fullest extent, must not only acquaint himself with symptoms and remedies, but with the many causes of diseases; with the laws of hygiene, and he must be capable of teaching his patrons why the observance of these laws result in health, and why violations of them bring disease and death. The physician cannot occupy the exalted station it is the privilege of his science to bestow, unless he shall honor that science by making himself familiar with the measures which prevent disease, as well as the causes which produce, and remedies which are intended to relieve them. It will be our object, in this department, to furnish, monthly, many facts, items and hints to aid the old as well as the young practitioner in the discharge of their duties as professional gentlemen, and guardians of the public health. We hope to furnish many things which will aid in the skillful management of disease at the bed-side and not unfrequently to prevent its occurrence, which is the great mission of the true Physician. It is expected of the Physician that he shnll be a man of varied and extensive information upon all topics legitimately connected with his profession. Where so much is expected, he should at least exert himself to become acquainted with the common laws of hygiene, so as to meet, to some extent, the demands of the public upon him.

---

